# The Type a and Type b Polysaccharide Capsules Predominate in an International Collection of Invasive *Kingella kingae* Isolates

**DOI:** 10.1128/mSphere.00060-17

**Published:** 2017-03-15

**Authors:** Eric A. Porsch, Kimberly F. Starr, Pablo Yagupsky, Joseph W. St. Geme

**Affiliations:** a Department of Pediatrics, The Children's Hospital of Philadelphia, Philadelphia, Pennsylvania, USA; b Department of Pediatrics, Duke University Medical Center, Durham, North Carolina, USA; c Department of Molecular Genetics and Microbiology, Duke University Medical Center, Durham, North Carolina, USA; d Soroka University Medical Center, Ben-Gurion University of the Negev, Beer-Sheva, Israel; e University of Pennsylvania Perelman School of Medicine, Philadelphia, Pennsylvania, USA; University of Kentucky

**Keywords:** *Kingella kingae*, PCR, capsule typing, clinical isolates, polysaccharide capsule

## Abstract

*Kingella kingae* is an encapsulated Gram-negative bacterium and an important etiology of osteoarticular infections in young children. A recent study examining a diverse collection of carrier and invasive *K. kingae* isolates from Israel revealed four distinct polysaccharide capsule types. In this study, to obtain a global view of *K. kingae* capsule type diversity, we examined an international collection of isolates using a multiplex PCR approach. The collection contained all four previously identified capsule types and no new capsule types. Over 95% of invasive isolates in the collection were type a or type b, similar to the findings in Israel. These results suggest that the type a and type b polysaccharide capsules may have enhanced pathogenic properties or may mark clonal groups of strains with specific virulence genes. In addition, they raise the possibility that a vaccine containing the type a and type b capsules might be an effective approach to preventing *K. kingae* disease.

**IMPORTANCE** *Kingella kingae* has emerged as a significant cause of septic arthritis, osteomyelitis, and bacteremia in young children. A recent study examining a diverse collection of *K. kingae* isolates from Israel revealed four different polysaccharide capsule types in this species, designated types a to d. To determine the global distribution of *K. kingae* capsule types, we assembled and capsule typed an international collection of *K. kingae* isolates. The findings reported here show that the type a and type b capsules represent >95% of the invasive isolates, similar to the Israeli isolate collection, suggesting that a polysaccharide-based vaccine targeting these two capsules could be an attractive approach to prevent *K. kingae* disease.

## Abstract (precis)

*Kingella kingae* has emerged as a significant cause of septic arthritis, osteomyelitis, and bacteremia in young children. A recent study examining a diverse collection of *K. kingae* isolates from Israel revealed four different polysaccharide capsule types in this species, designated types a to d. To determine the global distribution of *K. kingae* capsule types, we assembled and capsule typed an international collection of *K. kingae* isolates. The findings reported here show that the type a and type b capsules represent >95% of the invasive isolates, similar to the Israeli isolate collection, suggesting that a polysaccharide-based vaccine targeting these two capsules could be an attractive approach to prevent *K. kingae* disease.

## OBSERVATION

The use of improved culture techniques and PCR-based diagnostics in recent years has revealed that the Gram-negative bacterium *Kingella kingae* is a significant etiology of osteoarticular infections in children 6 to 48 months of age in countries where these sensitive detection methods are routinely employed (1). *K. kingae* is a normal component of the upper respiratory tract flora in young children and is present in the posterior pharynx in approximately 10% of healthy children 6 to 48 months of age at any given point in time (2–5). This organism is readily transmitted from person to person by close contact among young children, and longitudinal studies have estimated that children have an approximately 70% chance of being colonized with *K. kingae* during the first 2 years of life (3, 6).

In most individuals, colonization with *K. kingae* persists for weeks to months and is then cleared (3, 6). On occasion, the organism is able to breach the respiratory epithelial barrier, enter the bloodstream, and disseminate to distant sites, causing invasive disease. Analysis of the *K. kingae* population structure suggests that only some *K. kingae* strains are able to cause invasive disease (7). The primary clinical presentations of *K. kingae* disease include septic arthritis, osteomyelitis, spondylodiscitis, tenosynovitis, bacteremia without a focus, and endocarditis (8). The annual incidence of culture-proven disease among children younger than 5 years of age in Israel is 9.4 per 100,000. However, given the difficulty in cultivating *K. kingae*, this figure represents a minimal estimate (9). Recognizing that the use of species-specific nucleic acid amplification improves the detection of *K. kingae* by 500% compared to its detection by culture, the true incidence of *K. kingae* invasive disease in the Israeli population is likely comparable to the incidence of invasive *Haemophilus influenzae* type b disease before the introduction of *H. influenzae* type b conjugate vaccines (Hib conjugate vaccines) (i.e., >50 per 100,000) (10). The incidence of *K. kingae* invasive disease in other countries has not been defined but appears to be high in parts of Europe and a number of other countries around the world.

Recent studies have shown that isolates of *K. kingae* elaborate a polysaccharide capsule (11–13). Interestingly, elimination of encapsulation results in attenuated virulence in an infant rat model of invasive disease (14), suggesting that the capsule is an important virulence factor. Although the specific mechanism by which the capsule facilitates invasive disease has not been defined, the polysaccharide capsules of other pathogenic organisms prevent phagocytosis and block complement-mediated serum killing, promoting bacterial survival in the host. Given this information and the widespread success of polysaccharide conjugate vaccines in reducing morbidity and mortality due to a variety of encapsulated pathogenic bacteria, we recently defined the polysaccharide capsule repertoire in a diverse set of >400 Israeli *K. kingae* isolates (13). We found that four distinct polysaccharide capsule structures (capsule types a, b, c, and d) were present in this collection and that >95% of invasive disease isolates expressed the type a or type b capsule. Furthermore, we identified the *csa*, *csb*, *csc*, and* csd* capsule synthesis loci that are necessary for the expression of the type a, type b, type c, and type d capsules, respectively.

To gain a broader view of capsule type diversity in the global *K. kingae* population, in this study, we assembled an international collection of *K. kingae* isolates and determined the capsule type of each isolate using a multiplex PCR approach. We found that the same four capsule types identified in the Israeli isolate collection were present in the international collection and that no new capsule types were present. In addition, we established that over 95% of invasive isolates expressed the type a or type b capsule.

**FIG 1:**
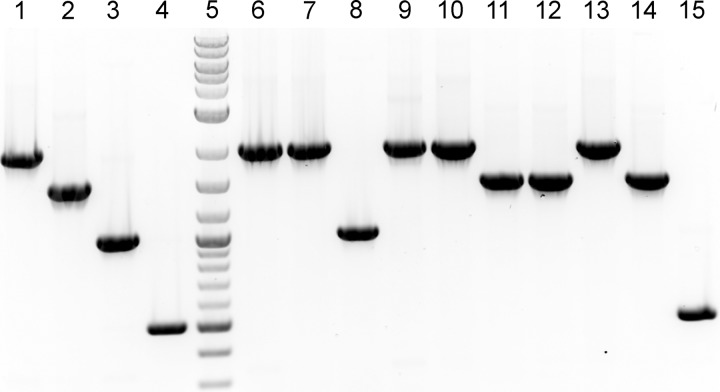
International *K. kingae* isolates are represented by four capsule types. (A) A representative agarose gel of the multiplex capsule-typing PCR approach is shown. The type a amplicon is ~2,000 bp (lane 1), the type b amplicon is ~1,500 bp (lane 2), the type c amplicon is ~1,000 bp (lane 3), and the type d amplicon is ~500 bp (lane 4). The genomic DNA PCR template sources are as follows: lane 1, KK01 (type a control); lane 2, KK58 (type b control); lane 3, KK60 (type c control); lane 4, BB270 (type d control); lane 5, DNA ladder; lane 6, 16RZ2819K (type a); lane 7, 16SB9163M (type a); lane 8, ATCC 23330 (type c); lane 9, ATCC 23331 (type a); lane 10, ATCC 23332 (type a); lane 11, SW353 (type b); lane 12, SW628 (type b); lane 13, SW268 (type a); lane 14, AUS 01 (type b); lane 15, KK194 (type d).

In a previous study, we developed a *K. kingae* capsule type PCR screening approach that relied on the use of five separate reactions: reactions specific to each of the four capsule types and a reaction that used primers flanking the capsule synthesis locus (13). To streamline the *K. kingae* capsule-typing process, in this work, we developed a single multiplex PCR approach for one-step identification of the capsule type. The reaction mixture contains four sets of primer pairs specific to each of the four capsule synthesis loci, designed to each produce a different size amplicon. As shown by the results in Fig. 1, the multiplex PCR produces an ~2,000-bp amplicon for the *csa* locus (encoding the synthesis gene for the type a capsule), an ~1,500-bp amplicon for the *csb* locus (encoding the synthesis genes for the type b capsule), an ~1,000-bp amplicon for the csc locus (encoding the synthesis genes for the type c capsule), and an ~500-bp amplicon for the *csd* locus (encoding the synthesis genes for the type d capsule).

**FIG 2:**
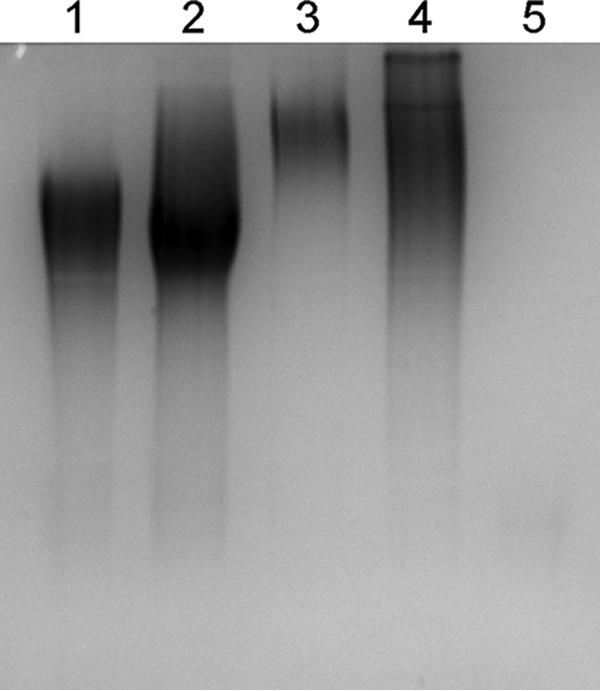
Isolate CAN8 is nonencapsulated. Mild-acid surface extracts from capsule type a strain KK01 (lane 1), type b strain KK58 (lane 2), type c strain KK60 (lane 3), type d strain BB270 (lane 4), and isolate CAN8 (lane 5) were analyzed following SDS-PAGE and alcian blue staining. The lack of alcian blue-stained material in the extract of CAN8 confirms that this isolate is nonencapsulated.

To investigate global capsule type diversity in* K. kingae*, we assembled an international strain collection consisting of 150 isolates (Table 1). Genomic DNA was recovered from each isolate and was used as the template in the multiplex PCR assay. We hypothesized that any isolate that failed to produce a capsule locus-specific amplicon could potentially contain a novel capsule synthesis locus or could be a nonencapsulated strain. In total, 89 isolates were capsule type a (59.3%), 49 isolates were type b (32.7%), 8 isolates were type c (5.3%), 3 isolates were type d (2.0%), and 1 isolate (0.7%) yielded no PCR product. Further examination of the isolate that yielded no PCR product revealed that it was nonencapsulated, as assessed by alcian blue staining of surface extracts and inspection of colony morphology (Fig. 2). Using PCR primers that flank the capsule synthesis locus, a 2.5-kb product was amplified from this isolate. Sequencing of this product revealed a truncated *csa* locus (data not shown). Accordingly, for the purposes of this study, this isolate was considered capsule type a.

**TABLE 1:** International isolate collection used in this study

**Isolate**	**Location**	**Year**	**Syndrome**^a^	**Capsule type**
CA7	Catalonia, Spain	1997	OA	a
CA20	Catalonia, Spain	1998	OA	a
CA40	Catalonia, Spain	2000	OA	b
CA48	Catalonia, Spain	2001	OA	b
CA49	Catalonia, Spain	2001	OA	b
CA55	Catalonia, Spain	2001	OA	c
CA57	Catalonia, Spain	2002	OA	a
CA61	Catalonia, Spain	2003	OA	a
CA63	Catalonia, Spain	2004	B	a
CA64	Catalonia, Spain	2004	B	a
CA65	Catalonia, Spain	2004	B	b
CA66	Catalonia, Spain	2004	OA	a
CA67	Catalonia, Spain	2004	B	b
CA68	Catalonia, Spain	2004	OA	a
CA73	Catalonia, Spain	2006	OA	b
CA75	Catalonia, Spain	2006	B	b
CA77	Catalonia, Spain	2006	OA	a
CA78	Catalonia, Spain	2006	OA	a
CA83	Catalonia, Spain	2007	OA	a
CA84	Catalonia, Spain	2007	OA	a
CA85	Catalonia, Spain	2007	OA	b
CA88	Catalonia, Spain	2008	OA	a
CA94	Catalonia, Spain	2008	OA	a
CA95	Catalonia, Spain	2008	OA	a
CA99	Catalonia, Spain	2009	OA	b
CA105	Catalonia, Spain	2009	B	a
CA112	Catalonia, Spain	2009	OA	a
CA151	Catalonia, Spain	2009	OA	b
CA179	Catalonia, Spain	2010	OA	a
CA120	Catalonia, Spain	2010	B	a
CA122	Catalonia, Spain	2010	OA	b
CA129	Catalonia, Spain	2011	OA	a
CA131	Catalonia, Spain	2011	OA	c
CA138	Catalonia, Spain	2012	OA	a
CA139	Catalonia, Spain	2012	C	d
CA181	Catalonia, Spain	2014	OA	b
CA183	Catalonia, Spain	2014	C	a
CA189	Catalonia, Spain	2014	OA	a
CA197	Catalonia, Spain	2014	OA	a
CA198	Catalonia, Spain	2014	OA	b
CA199	Catalonia, Spain	2014	OA	b
CA202	Catalonia, Spain	2015	OA	a
CA203	Catalonia, Spain	2015	OA	b
CA216	Catalonia, Spain	2016	B	a
CA217	Catalonia, Spain	2016	OA	b
CIP 73.01	Besançon, France	1972	B	a
CIP 101722	Grenoble, France	1985	B	a
CIP 102473	Paris, France	1986	OA	b
SCH 25972	Paris, France	2007	OA	b
BIA 29991	Paris, France	2009	OA	a
SAN 6539	Bordeaux, France	2009	OA	b
LEP 6724	Bordeaux, France	2009	UN^b^	b
BOU 30672	Paris, France	2010	OA	a
N10-6602	Nantes, France	2010	OA	a
N10-6318	Nantes, France	2010	OA	a
HER 31223	Paris, France	2010	OA	a
N10-10770	Nantes, France	2010	B	d
GRO 7183	Bordeaux, France	2010	OA	a
N10-10419	Nantes, France	2010	EC	b
ETI 126580	Paris, France	2011	OA	a
HAM 137138	Paris, France	2011	OA	b
SAI 11985	Paris, France	2011	OA	a
POH 14284	Paris, France	2011	OA	a
MAR 1853	Paris, France	2011	OA	a
STF 4A	Paris, France	2011	C	a
DER 112012–1	Paris, France	2012	OA	a
NICE 476	Nice, France	2012	OA	b
DOR 8225	Bordeaux, France	2012	UN	a
CAT30640171-S	Paris, France	2013	OA	b
COU 1310053120	Sables d’Olonnes, France	2013	EC	a
FOF 3022006-S	Paris, France	2013	OA	b
DAG 31560001-S	Paris, France	2013	OA	a
ZEH 30720143-S	Paris, France	2013	OA	a
BRU32800001LA	Paris, France	2013	OA	a
KWG-1	Paris, France	2013	OA	a
AUD31930140-S	Paris, France	2013	OA	a
BON 36648-la	Paris, France	2013	OA	b
ZUL 30220039-S	Paris, France	2013	C	b
CRA 32950107-S	Paris, France	2013	UN	a
AGO 30220109-S	Paris, France	2013	OA	a
M2003000170	Minnesota, USA	2003	C	a
C2003003154	Minnesota, USA	2003	OA	a
M2004000037	Minnesota, USA	2004	C	b
C2005003818	Minnesota, USA	2005	B	a
C2005004024	Minnesota, USA	2005	B	a
C2005004457	Minnesota, USA	2005	OA	b
C2005004524	Minnesota, USA	2005	OA	b
C2006001196	Minnesota, USA	2006	OA	b
C2006001744	Minnesota, USA	2006	OA	a
C2006003006	Minnesota, USA	2006	B	a
C2007000490	Minnesota, USA	2007	OA	b
Duke 137	North Carolina, USA	2007	EC	a
C2009000170	Minnesota, USA	2009	B	b
C2009033016	Minnesota, USA	2009	OA	a
C2012000896	Minnesota, USA	2012	OA	a
13040	Pennsylvania, USA	2016	OA	a
97–982	Missouri, USA	UN	OA	a
SLCH 05-001-1817	Missouri, USA	UN	OA	a
SLCH 002	Missouri, USA	UN	OA	b
PP1	New York, USA	UN	OA	a
VTK:500285	USA	UN	UN	c
VTK:500287	USA	UN	UN	c
VTK:501585	USA	UN	UN	a
VTK:501586	USA	UN	UN	c
VTK:500615	USA	UN	UN	a
VTK:501628	USA	UN	UN	a
VTK:501629	USA	UN	UN	a
VTK:501804	USA	UN	UN	a
CAN1	Montreal, Canada	2003	OA	a
CAN2	Montreal, Canada	2005	OA	a
CAN3	Montreal, Canada	2005	OA	a
CAN7	Montreal, Canada	2006	OA	b
CAN8^c^	Montreal, Canada	2007	OA	a
CAN9	Montreal, Canada	2007	OA	a
CAN13	Montreal, Canada	2009	OA	b
CAN16	Montreal, Canada	2010	OA	b
CAN21	Montreal, Canada	2012	OA	b
CAN22	Montreal, Canada	2012	B	b
CAN24	Montreal, Canada	2013	OA	a
CAN25	Montreal, Canada	2013	OA	a
9508+31135	Reykjavíc, Iceland	1995	OA	b
9911+17199	Reykjavíc, Iceland	1999	OA	a
0111+28183	Reykjavíc, Iceland	2001	OA	b
0211+12480	Reykjavíc, Iceland	2002	OA	b
0303+28260	Reykjavíc, Iceland	2003	OA	b
0309+30201	Reykjavíc, Iceland	2003	OA	c
0405+30002	Reykjavíc, Iceland	2004	OA	b
0410+23083	Reykjavíc, Iceland	2004	B	a
0601+26281	Reykjavíc, Iceland	2006	OA	a
0604+12258	Reykjavíc, Iceland	2006	OA	b
S0910230213	Reykjavíc, Iceland	2009	OA	b
S1010080184	Reykjavíc, Iceland	2010	OA	c
09WG5552P	Christchurch, New Zealand	2009	B	a
09WT1836F	Christchurch, New Zealand	2009	B	a
11DC5983H	Christchurch, New Zealand	2011	B	a
15JS24141R	Christchurch, New Zealand	2015	B	a
15RB7013L	Christchurch, New Zealand	2015	OA	a
15RJ0022G	Christchurch, New Zealand	2015	OA	a
15R43594M	Christchurch, New Zealand	2015	C	a
16RZ0774E	Christchurch, New Zealand	2016	C	a
16RZ2819K	Christchurch, New Zealand	2016	C	a
16SB9163M	Christchurch, New Zealand	2016	C	a
ATCC 23330	Norway	1960s	C	c
ATCC 23331	Norway	1960s	B	a
ATCC 23332	Norway	1960s	B	a
SW353	Switzerland	2013	C	b
SW628	Switzerland	2014	OA	b
SW268	Switzerland	2015	OA	a
AUS 01	Geelong, Australia	2013	EC	b
KK194	St. Petersburg, Russia	2003	C	d

a B, bacteremia; C, carriage; EC, endocarditis; OA, osteoarticular.

b UN, unknown.

c Isolate CAN8 is nonencapsulated due to a truncated *csaA* gene but was included as a type a isolate for the purposes of this analysis.

A detailed summary of the *K. kingae* capsule-typing results by country and syndrome (invasive isolate, carrier isolate, or unknown) at the time of isolation is shown in Table 2. Among the 126 isolates that were known to be recovered from systemic sites in patients with invasive *K. kingae* disease (osteoarticular infections, bacteremia, or endocarditis), 76 were type a (60.3%), 45 were type b (35.7%), 4 were type c (3.2%), and 1 was type d (0.8%), similar to the breakdown among invasive isolates in Israel (13). Of the 13 carrier isolates from oropharyngeal swabs, 7 were type a (53.8%), 3 were type b (23.1%), 1 was type c (7.7%), and 2 were type d (15.4%). While the small number of carrier isolates precludes any meaningful analysis of capsule type distribution, the relative representation of each type was similar to the distribution in the previously published Israeli carrier isolate collection (49.0% type a, 19.2% type b, 12.1% type c, and 19.7% type d) (13). With regard to the countries with at least 10 isolates, >80% of isolates were type a or type b, and in all countries except Iceland, the most common capsule type was type a. Interestingly, all 10 of the New Zealand isolates were type a, including the 4 carrier isolates.

**TABLE 2:** Summary of capsule-typing results based on country of isolation and associated clinical condition

Country (no. of isolates)	No. of isolates from indicated clinical condition with indicated capsule type														
	Invasive					Carrier					Unknown				
	Total no.	a	b	c	d	Total no.	a	b	c	d	Total no.	a	b	c	d
Spain (45)	43	25	16	2		2	1			1					
France (36)	30	20	9		1	2	1	1			3	2	1		
USA (28)	18	12	6			2	1	1			8	5		3	
Canada (12)	12	7^a^	5												
Iceland (12)	12	3	7	2											
New Zealand (10)	6	6				4	4								
Norway (3)	2	2				1			1						
Switzerland (3)	2	1	1			1		1							
Australia (1)	1		1												
Russia (1)						1				1					
Total (150)	126	76	45	4	1	13	7	3	1	2	11	7	1	3	

a Isolate CAN8 is nonencapsulated due to a truncated *csaA* gene but was included as a type a isolate for the purposes of this analysis.

The four previously identified *K. kingae* capsule types that were originally characterized in a diverse collection of Israeli isolates were also identified in the international collection of isolates presented here. No new capsule types were identified in this international collection, confirming the limited capsular repertoire of *K. kingae* compared to those of other pathogens that reside in the respiratory tract, such as *Neisseria meningitidis* (13 capsule types) and *Streptococcus pneumoniae* (over 90 capsule types). The study has the limitation that carrier isolates were poorly represented, reflecting the lack of *K. kingae* colonization studies using culture-based methods outside Israel. As a consequence, we cannot exclude the possibility that other capsule types or nonencapsulated strains exist in the countries examined in this study, keeping in mind the likelihood that some *K. kingae* strains are able to colonize the oropharynx but are unable to cause invasive disease. In addition, there is little information on the epidemiology of *K. kingae* disease in developing countries, which may harbor unidentified capsule types. Another limitation of this study is the lack of complete epidemiological data on every isolate. Therefore, the capsule type distribution may be skewed due to the presence of epidemiologically related strains in this collection.

Given that the capsule-typing multiplex PCR approach is specific for the four known *K. kingae* capsule types, we anticipated that new capsule types in this international collection of isolates would be associated with the lack of a PCR amplicon. The only isolate that failed to yield an amplicon in the multiplex PCR was strain CAN8. Using PCR primers that flank the capsule synthesis locus, we were able to amplify a 2.5-kb amplicon from this strain. Nucleotide sequencing of this amplicon revealed a truncated *csaA* gene with a large, 1,085-bp deletion in the 3′ region, resulting in a nonencapsulated phenotype. Thus, CAN8 is a type a strain that presumably experienced a deletion event and does not have a novel capsule type.

In conclusion, we found that 95.1% of the international invasive disease isolates were either capsule type a or b, virtually identical to the 96.0% of Israeli invasive disease isolates that were either type a or type b (13). This finding is similar to the situation with *H. influenzae*, where isolates expressing the serotype b capsule were responsible for >95% of all invasive *H. influenzae* disease in the pre-Hib vaccine era, despite the presence of 6 different polysaccharide capsule types (capsule types a to f) (15). It is possible that the *K. kingae* type a and type b polysaccharide capsules have enhanced pathogenic properties. Alternatively, these capsules may be associated with clonal groups of strains that harbor important virulence genes. Because anticapsular antibody protects against disease caused by organisms such as *H. influenzae* type b and *S. pneumoniae*, it is intriguing to speculate that a type a-type b capsular polysaccharide conjugate vaccine might be an effective strategy to prevent disease by *K. kingae*.

## 

### Bacterial strains.

The international *K. kingae* isolates examined in this study are listed in Table 1. The capsule type a (KK01), capsule type b (KK58), capsule type c (KK60), and capsule type d (BB270) control strains were described previously (13). The *K. kingae* isolates were grown on chocolate II agar (BD, Sparks, MD) for 17 to 18 h at 37°C in a humidified 5% CO_2_ atmosphere. The isolates were stored in brain heart infusion (BHI) broth with 20% glycerol at −80°C.

### Capsule typing.

A multiplex PCR strategy was developed to screen for the presence of each of the four unique capsule synthesis loci in a single PCR assay. The primers were designed to produce an ~2,000-bp amplicon for the *csa* locus (*csa*multiF, 5′ AGTACAGAACACTTGTTGTTGC 3′, and *csa*multiR, 5′ AACATTGGCGCAGACAAATTC 3′), an ~1,500-bp amplicon for the *csb* locus (*csb*multiF, 5′ AGATTGGTGGACTTTATATGGTAATTATG 3′, and *csb*multiR, 5′ AAATAGAATATTGCGACTGTGCG 3′), an ~1,000-bp amplicon for the *csc* locus (*csc*multiF, 5′ CATTAGCATTGATGCCATTTATGAAC 3′, and *csc*multiR, 5′ CGATTGATGACTATTAAACCTTCGG 3′), and an ~500-bp amplicon for the *csd* locus (*csd*multiF, 5′ AAAGGTAAATATCAATTTGCAATTATTTGC 3′, and *csd*multiR, 5′ CTTAATAGGACATCATCACCCAAATC 3′). Genomic DNA from the *K. kingae* isolates was prepared using the Wizard genomic DNA purification kit (Promega, Madison, WI). Each PCR mixture contained 5 μl of 2.0× *Taq* red Apex master mix (Genesee Scientific, San Diego, CA), 0.5 μl of genomic DNA template, each of the eight primers at a final concentration of 125 nM, and sterile PCR-grade H_2_O in a total reaction mixture volume of 10 μl. The cycling conditions were as follows: 2 min at 94°C, 30 cycles of 15 s at 94°C, 20 s at 58°C, and 1 min at 72°C, and a final 5-min extension at 72°C. Three microliters of each PCR mixture was analyzed using agarose gel electrophoresis, and the capsule type was determined by the size of the amplicon (Fig. 1). The accuracy of this multiplex PCR strategy in determining capsule type was confirmed by comparing the results with the previously published results of the original PCR-based capsule-typing system (13).

### Alcian blue staining.

Surface extracts were prepared using Tris-acetate, pH 5.0, separated using SDS-PAGE, and stained with alcian blue as described previously (11).
